# Double-layer symmetric gratings with bound states in the continuum for dual-band high-*Q* optical sensing

**DOI:** 10.3762/bjnano.13.116

**Published:** 2022-11-25

**Authors:** Chaoying Shi, Jinhua Hu, Xiuhong Liu, Junfang Liang, Jijun Zhao, Haiyan Han, Qiaofen Zhu

**Affiliations:** 1 School of Mathematics and Physics Science and Engineering, Hebei University of Engineering, Handan, Hebei 056038, P. R. China; 2 School of Information and Electrical Engineering, Hebei University of Engineering, Handan, Hebei 056038, P. R. China

**Keywords:** bound states in the continuum, dual band, high quality factor, localized optical field, nonlinear optics, optical sensing

## Abstract

Herein, we theoretically demonstrate that a double-layer symmetric gratings (DLSG) resonator consisting of a low-refractive-index layer sandwiched between two high-contrast gratings (HCG) layers, can host dual-band high-quality (*Q*) factor resonance. We find that the artificial bound states in the continuum (BIC) and Fabry–Pérot BIC (FP-BIC) can be induced by optimizing structural parameters of DLSG. Interestingly, the artificial BIC is governed by the spacing between the two rectangular dielectric gratings, while the FP-BIC is achieved by controlling the cavity length of the structure. Further, the two types of BIC can be converted into quasi-BIC (QBIC) by either changing the spacing between adjacent gratings or changing the distance between the upper and lower gratings. The simulation results show that the dual-band high-performance sensor is achieved with the highest sensitivity of 453 nm/RIU and a maximum figure of merit (FOM) of 9808. Such dual-band high-*Q* resonator is expected to have promising applications in multi-wavelength sensing and nonlinear optics.

## Introduction

High quality (*Q*) factor resonance in nanophotonics has attracted considerable attention in the past decades due to its wide applications in narrow-band filters [1], nonlinear optics [2], optical sensors [3] and lasers [4]. To date, most researchers have focused their interests on the single high-*Q* resonance of various structures and proposed different types of structures to achieve high-*Q*-factors, such as metallic structures based on surface plasmon resonances [5–6], Mie resonance-based dielectric structures [7–8], and high-contrast grating (HCG) structures in periodic subwavelengths [9–10]. Among them, HCG structures built on silicon-on-insulator (SOI) substrates establish a new platform for integrated optics as well as optical sensing [11–12] owing to its high reflectivity in bandwidth (>99%) and compatibility with the complementary metal oxide semiconductor (CMOS) processes [13–14]. It has been shown that the HCG system can support the optical bound states in the continuum (BICs) [15–18]. BIC plays an important role in determining the characteristics of the radiative high-*Q* resonance [17]. However, there are fewer reported HCG structures that support dual-band high-*Q* resonances. Differing from a single high-*Q* resonance, dual-band high-*Q* resonances allows for simultaneous modification of the line shape at two spectral locations [19], which provides multiple detection points for sensing applications.

In 1929, von Neumann and Wigner first proposed the BIC theory shortly after the advent of quantum mechanics [20], which was then extended to acoustics, electromagnetism, and other fields [21–24]. A true BIC has an infinite *Q*-factor and vanishing resonant linewidth, and this can only exist in an ideal lossless infinite structure or in extreme values of the parameters [25–26]. The structures that are commonly used to induce the BIC include metasurfaces [27–28], dielectric gratings [29–30], photonic crystals [31], and whispering-gallery resonators [32]. In 2016, Wang et al. investigated a symmetry-protected BIC (SP-BIC) supported by a slotted HCG structure in both TE and TM polarization scenarios [17]. When the spatial symmetry of the mode is incompatible with the symmetry of the outgoing wave, the coupling coefficient vanishes thus inducing the SP-BIC. In 2019, Doskolovich et al. reported that a Fabry–Pérot BIC (FP-BIC) can be excited by varying the distance of the same grating ridge on the surface of a single-mode dielectric slab waveguide [33]. Generally speaking, an FP-BIC can be formed when the spacing between two resonances is changed so that the sum of the round-trip phase shifts is an integer multiple of 2π. In 2020, Lee et al. showed that photonic lattices with a symmetric cladding structure support Friedrich–Wintgen BIC (FW-BIC), which occurs due to the destructive interference of two resonances coupled to the same radiation channel [18]. From a practical application perspective, the BIC must be converted to quasi-BIC (QBIC) with a finite *Q*-factor or the full width at half maximum (FWHM) so they can be accessed by an external excitation, such as a plane wave [34–35]. QBIC has been extensively utilized in optical absorbers [36–37], lasers [38], filters [30], and sensors [39]. Particularly, the QBIC sensor enables highly accurate detection of environmental changes by reading variations in the spectrum. However, numerous research works have focused on the BIC mechanism of single-mode resonance [26,28], which may limit its application.

In this work, we proposed a double-layer symmetric gratings (DLSG) structure supporting artificial BIC and FP-BIC, which is composed of highly reflecting HCG layers surrounding a low refractive index layer. The artificial BIC was excited by tuning the spacing between two adjacent dielectric gratings. More importantly, we found that the modes can be evolved by controlling the grating gaps. Then, the FP-BIC is induced by varying the cavity length of the structure. Finally, we achieved a dual-band high-*Q* resonator for optical sensing, which is potentially valuable for applications in multi-wavelength sensing.

## Structure and Principle

**Figure 1:**
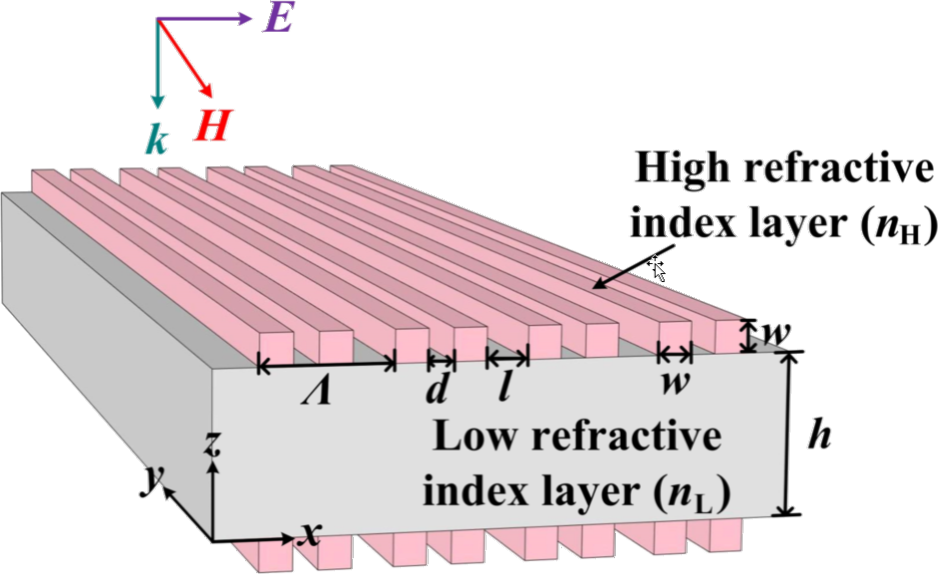
Schematic of the double-layer symmetric gratings structure.

The geometry of our designed structure is shown in Figure 1, where the high refractive index rectangular dielectric gratings (RDGs) are periodically arranged at the top/bottom of the low refractive index layer and are symmetrical. Besides, the structure possesses a translational period (Λ) along the *x*-direction and an infinite ridge along the *y*-direction. A unit cell contains two RDGs, and the spacing between the two RDGs in a period and not in a period are *d* and *l*, respectively. The side length of the RDG is *w* and the thickness of the low refractive layer is *h*. Moreover, the rigorous coupled-wave analysis (RCWA) [40–41] is used to design the structure of the device and analyze the spectral information, which is combined with the finite element method (FEM) [42] to compute the complex eigenfrequencies.

In order to investigate the resonance properties of dielectric gratings, we used here a dimensionless parameter α to measure the grating spacing of the DLSG structure by varying the distance between adjacent RDGs, which is defined as follows [26,43]:


[1] $$\alpha =\frac{d-l}{d+l}.$$

The complex eigenfrequencies of the DLSG structure were then obtained by the FEM, and can be described as *N* = ω − *i*γ, where ω and γ are the real and imaginary parts of *N*, respectively. The ω indicates the resonant frequency, and the γ refers to the radiative leakage of the electromagnetic energy stored in the leaky mode [44]. It allows expressing the radiative *Q*-factor in the following form:


[2] $$Q=\frac{\omega }{2\gamma }.$$

The BIC induced based on the structure of the HCG gratings can be divided into two categories, one is the BIC that is forbidden due to the coupling of symmetry with free space at normal incidence, and the other is the BIC that can occur by changing any structural parameter independent of the symmetry type [45]. In the proposed symmetric structure, we were able to find the artificial BIC by the Brillouin band folding phenomenon resulting from changing the spacing of the grating. Interestingly, the FP-BIC is obtained by varying the cavity length of the DLSG to satisfy the transverse resonance principle.

## Results and Discussion

As an example, the high refractive index layer of the structure depicted in Figure 1 is silicon (*n*_H_ = 3.47) and the low refractive index layer is SU-8 (*n*_L_ = 1.57). The other structural parameters are *w* = 200 nm, *h* = 1000 nm, and Λ = 800 nm. Besides, the whole structure is suspended in vacuum (*n*_c_ = 1.0) and normally incident by a TM-polarized plane wave (H//y).

**Figure 2:**
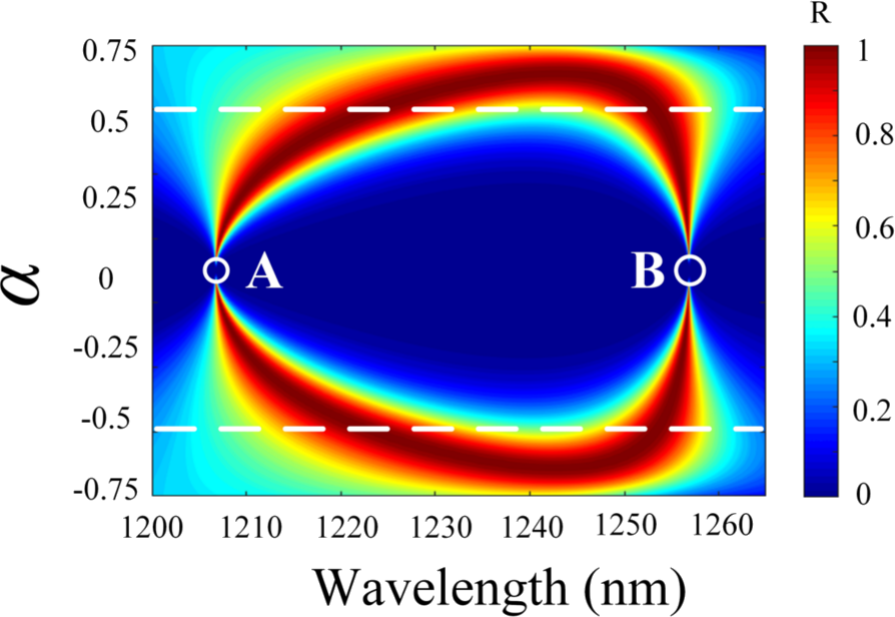
Reflection spectra mapping of the DLSG-based structure with respect to wavelength and α.

By varying the spacing *d* of the RDGs, the reflection spectra were obtained for resonances with different α values, as shown in Figure 2. The mode of the resonances can be regulated by controlling the spacing between the two RDGs, which is essentially a splitting of the simple mode. It can be seen from Figure 2 that the linewidth of the spectrum completely disappears when α = 0, which means that the *Q*-factor is infinite and the ideal BIC appears (points A and B). Furthermore, it is noticed that the evolution of the two modes is symmetric with respect to α = 0. Here, we take α > 0 as an example to analyze. The resonance evolves into two modes (referred to as the nonsimple state) when 0 < α < 0.5, and the QBIC near the points A and B possess a high *Q*-factor. When 0.5 < α < 0.75, the two resonances start to merge into a single one (referred to as the simple state), and the *Q*-factor is lower at this time. Therefore, the evolution of single and double resonances can be controlled by changing the spacing of RDGs. Moreover, changing the grating spacing allows the emergence of BIC.

**Figure 3:**
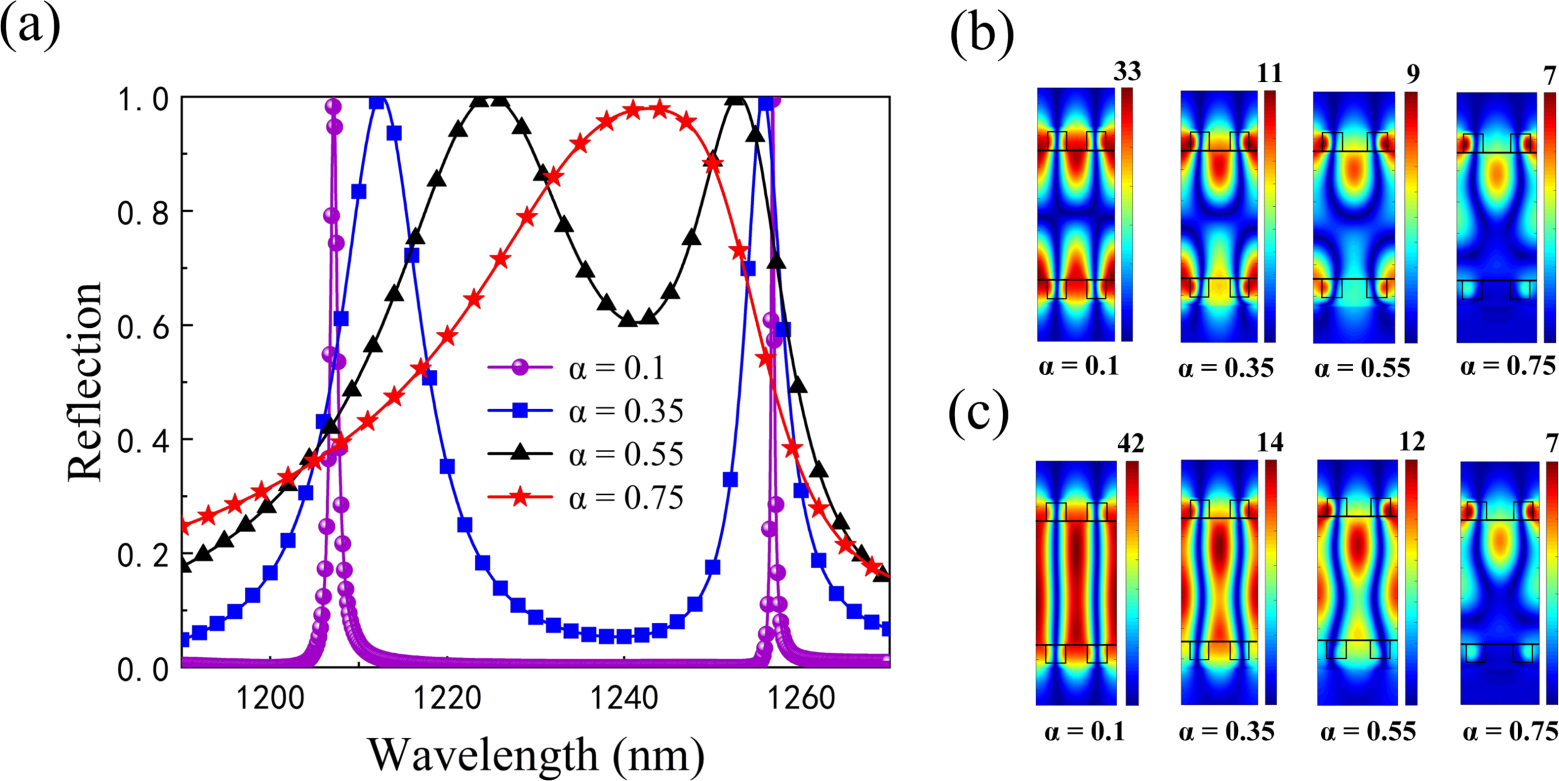
a) Reflection spectra of the structure with different α values. The magnetic field contours |*H*y| for b) mode 1 and c) mode 2 at different α values, respectively.

The reflection spectra calculated using RCWA when α = 0.1, 0.35, 0.55, 0.75 are given in Figure 3a, which shows in more detail the tuning of the RDGs spacing on single and double resonances. As α decreases, the linewidth of the resonance also decreases and evolves from the simple state to the nonsimple state until α = 0. At that time, the spectral linewidths of the two resonance peaks almost completely disappear and the ideal BIC is obtained. Furthermore, the magnetic field distributions of the two modes under resonance are calculated as shown in Figure 3b and Figure 3c. The localized field energy of both modes decreases as α increases, and the magnetic field distributions of both modes gradually converge to an identical one, thus achieving the simple merged state for the two modes (α = 0.75).

**Figure 4:**
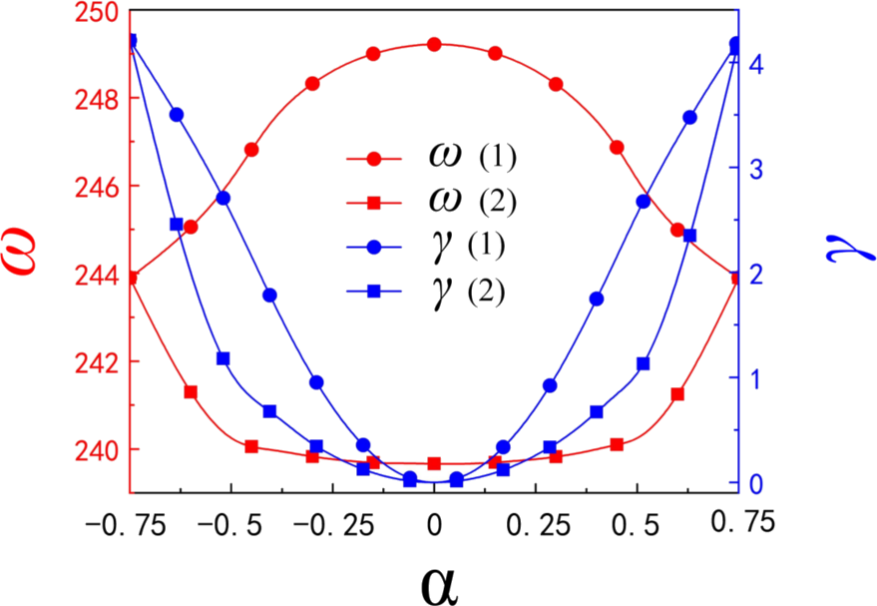
The complex eigenfrequencies of the two modes with respect to α.

Figure 4 shows the complex eigenfrequencies of the two modes at different α values. As seen in Figure 4, the ω value of mode 1 shows a tendency to increase and then it decreases as α grows; however, the trend of mode 2 is exactly the opposite. Moreover, the ω values of the two modes are symmetrical at α = 0. An increase of α from 0 to 0.75 implies the evolution of the two resonances, and the ω values of the two modes overlap when α = 0.75, realizing the simple state of the mode. When α = 0, the complex eigenfrequencies of mode 1 and mode 2 are 248.08 and 238.35 THz, respectively. In addition, it is apparent that the γ value of both modes are close to zero, which means that there is almost no radiation loss resulting in an infinite value for the *Q*-factor (the ideal BIC). Here, the spacing between each RDG is completely equal and exactly the same as the side length of the RDG (*d* = *l* = *w* = 200 nm), at which time the device is a periodic structure with a double-layer single grating. Moreover, the γ value of both modes increases with α, which leads to an increase in the radiation loss of the device and a decrease in the *Q*-factor. By physically varying the spacing of the RDG, the modes at the edge of the first Brillouin zone now lie at the gamma point (the center of the first Brillouin zone), thus resulting in an artificial BIC caused by the Brillouin folding phenomenon [45]. Such BIC is dominated by the electric toroidal dipole rather than the SP-BIC dominated by the magnetic dipole [46]. As a result, instead of having to break the symmetry of the structure, as in the case of SP-BIC, a simple change in the grating spacing can transform the BIC into a QBIC, leading to high-*Q* resonance.

**Figure 5:**
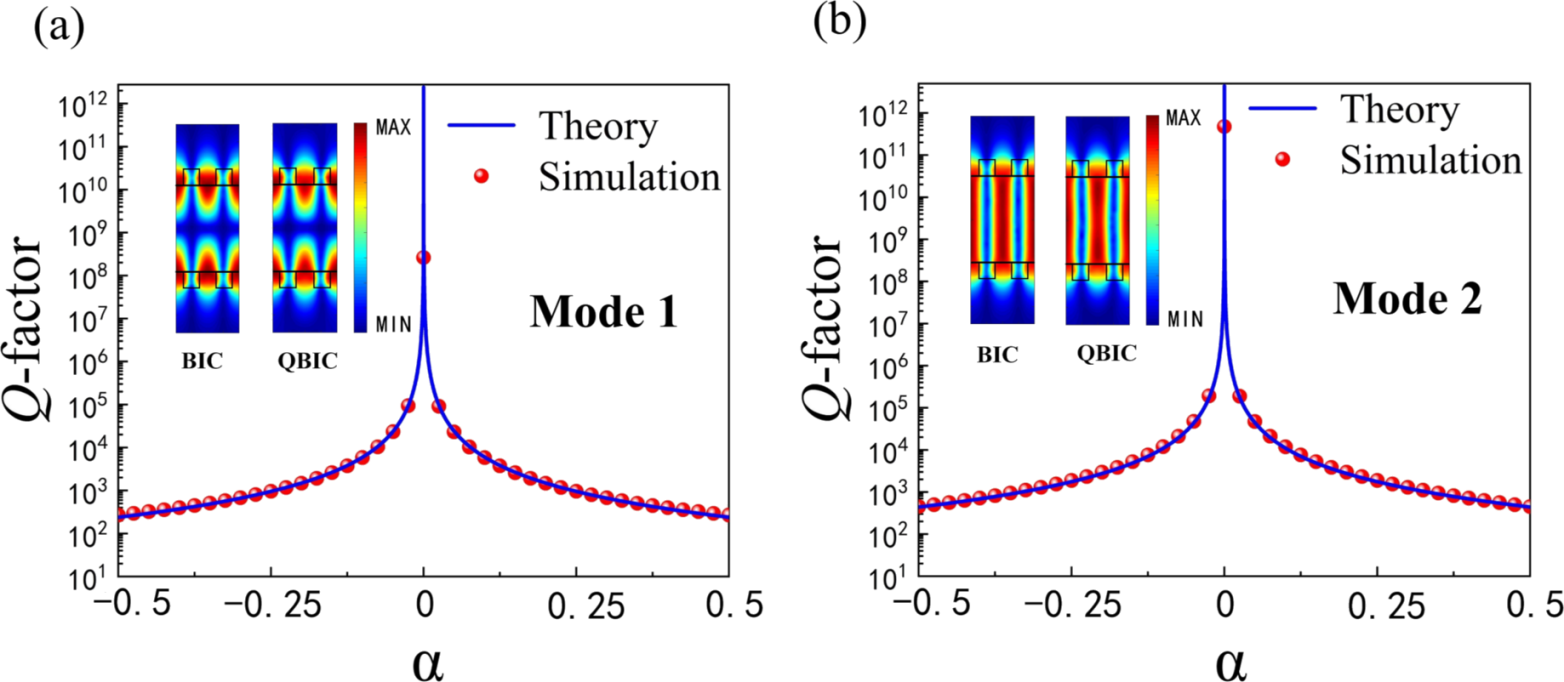
The *Q*-factor as a function of α of a) mode 1 and b) mode 2. The insets show the field distributions of the corresponding BIC and QBIC for the two modes.

The relationship between the radiative *Q*-factors calculated according to Equation 2 and the α values for both modes is investigated in Figure 5. It can be found that our calculated radiative *Q*-factor (dots) shows an inverse square dependence on α and agrees well with the theoretical prediction (*Q*_0_ = *C*α^−2^, solid line. Here, *C* is a constant determined by the design of the structure surface) [26]. In addition, the *Q*-factors of mode 1 and mode 2 are 2.6285 × 10^8^ and 4.7754 × 10^11^, respectively. It is noted that the *Q*-factor of mode 1 is always lower than that of mode 2 for the same value of α, which is attributed to different distributions of the localized optical field in the two modes. As seen in Figure 3b and Figure 3c, the optical field of mode 1 is mainly localized near the upper and lower gratings at the same α value, whereas most of the optical field in mode 2 is confined to the cavity. To physically obtain a high *Q*-factor, it is required that the optical field is trapped in a grating-based resonant cavity.

Theoretically, the BIC is a completely confined bound mode and cannot be excited [17]. However, the simulation results show that the artificial BIC can be transformed into a high-*Q* resonance with a *Q*-factor controlled by the spacing of the grating. The artificial BIC is perturbed by changing the grating spacing, and when overly perturbed (the spacing between the gratings is large) the two modes evolve into a simple mode with low-*Q* resonance. Notably, the high *Q*-factor appears in the near artificial BIC region (the insets in Figure 5), which is the region of enhanced field effects at energy points close to the BIC with limited but high *Q*-factors (QBIC) [47].

**Figure 6:**
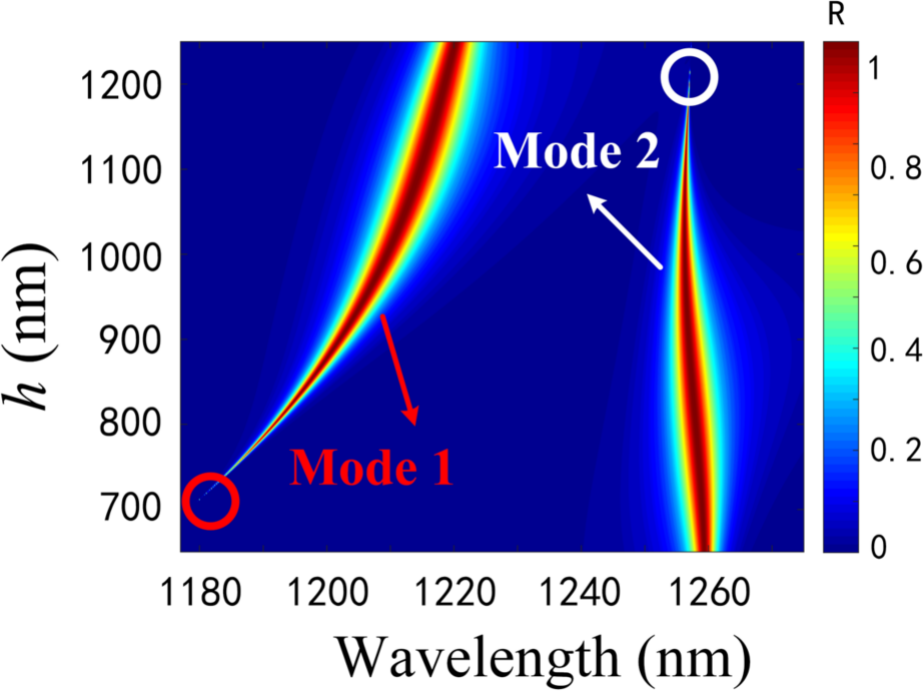
Reflection spectra mapping of the sensor with respect to wavelength and different *h* values at α = 0.15.

The change in spacing between RDGs can tune the evolution of the two modes and achieve artificial BIC. Then we fixed α and investigated the reflection spectra at different cavity lengths *h* by using RCWA. On the one hand, the two modes start to evolve into one mode when α > 0.5, and on the other hand, a smaller α value can ensure a higher *Q*-factor. Therefore, we fixed α = 0.15, and studied the reflection spectra for different cavity lengths at the same α in the presence of two modes, which is shown in Figure 6.

The FWHM of mode 1 increases with *h* and the spectrum almost disappears at approx. *h* = 700 nm (red circles). When *h* changes from 700 to 1200 nm, the spectrum of mode 2 gradually becomes narrower until it disappears (white circles). This implies that mode 1 and mode 2 can induce the BIC through the control of cavity length. Accordingly, the change of the cavity length enables the modulation of the two modes of FWHM for the purpose of tuning the *Q*-factor. If a system contains two resonances whose positions and FWHM can be changed by a physical parameter, then there may exist a value within a certain parameter range that will make one of the resonances become a bound state [48]. A single grating layer in the proposed structure is 100% reflective, and the two identical gratings at the top and bottom of the structure are equivalent to two identical parallel reflectors (separated by a spacing of *h*) in the Fabry–Pérot cavity model, where the modes trapped between the two identical gratings are decoupled from the continuum [49–50]. In other words, the resonance in the single-layer grating is split into an extremely narrow and a wide resonance in the double-layer gratings for a given cavity length *h*. Without external driving sources, the two resonance amplitudes *A* = (*A*_1_,*A*_2_)^T^ in the DLSG structure evolve in time as *i*∂*A*/∂*t* = *HA* with Hamiltonian [51–53]:


[3] $$H=\left[\begin{array}{cc} \omega_{0} & \kappa \\ \kappa & \omega_{0} \end{array}\right]-i\gamma_{0}\left[\begin{array}{cc} 1 & e^{i\psi }\\ e^{i\psi } & 1 \end{array}\right],$$

where κ is the near-field coupling between the two modes, ω_0_ and γ_0_ are the resonant frequency and radiation rate of the single grating resonance, respectively, and ψ is the propagation phase shift between the two resonators, satisfying the relation ψ = *kh*, where *k* is the transverse wave number. Then the two complex eigenfrequencies of *H* can be solved from Equation 3:


[4] $$\omega_{\pm }=\omega_{0}\pm \kappa -i\gamma_{0}\left(1\pm e^{i\psi }\right).$$

**Figure 7:**
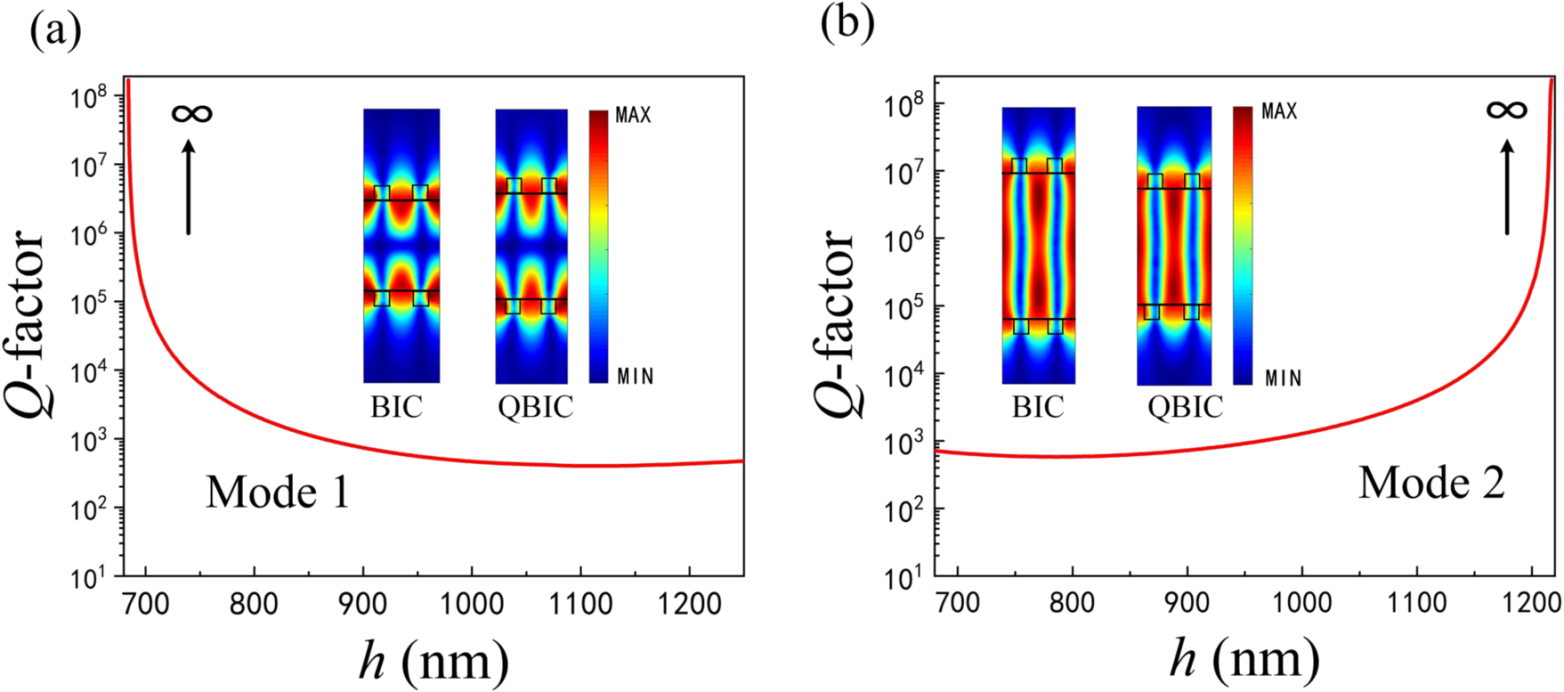
The *Q*-factor as a function of cavity length *h* of a) mode 1 and b) mode 2. The insets show the field distributions of the corresponding BIC and QBIC for the two modes.

Tuning the cavity length *h*, when the round-trip phase shift is 2*m*π (*m* = 0, 1, 2, …), the complex eigenfrequency of one mode in Equation 4 is ω_0_ ± κ – 2*i*γ with twice as much radiation loss as before. And the complex eigenfrequency of the other mode is ω_0_ ± κ with a pure real number, which indicates that it is an FP-BIC with an infinite *Q*-factor. The corresponding radiative *Q*-factors of the two modes at different *h* values when other structural parameters are constant are given in Figure 7. It can be found that mode 1 and mode 2 have infinite *Q*-factors at approx. 700 and 1200 nm, respectively. Consequently, both modes of the FP-BIC are governed by *h*. We can tune the linewidth and the corresponding *Q*-factor by changing the cavity length. The insets in Figure 7 show the optical field distributions of the ideal BIC and the QBIC corresponding to the two modes. It is clear that the QBIC near the ideal BIC also possesses a strong localized optical field. Therefore, the FP-BIC can be perturbed by the variation on the cavity length.

To further understand the capability of different cavity lengths of the two modes to confine the localized optical field, the energy ratio of the cavity to the unit cell is analytically calculated from the perspective of the complex eigenfrequencies, which is defined as follows [54]:


[5] $$\Gamma =\frac{\int_{C}{\left|H_{\text{norm}}\right|}^{2}\text{d}x\text{d}z}{\int_{U}{\left|H_{\text{norm}}\right|}^{2}\text{d}x\text{d}z},$$

where *C* and *U* represent the integration domain for the cavity and the unit cell, respectively. *H*_norm_ represents the magnetic field intensity distributions of the complex eigenfrequencies.

It can be concluded from Equation 5 that the larger the confinement factor Γ the stronger the capacity of the cavity to confine the optical field. The calculation results are presented in Table 1. With respect to mode 1, the increase in cavity length leads to a decrease in the confinement capacity of the cavity to light (Γ decreases from 0.5381 to 0.3782). On the contrary, since most of the light in mode 2 is trapped inside the cavity, the increase of *h* improves the confinement capacity of the cavity (Γ increases from 0.6344 to 0.7513). Here, the flexible choice of cavity length enables to compress the effective volume for the resonant mode, which results in an enhanced confinement of the cavity to the optical field and an improved *Q*-factor.

**Table 1:** Confinement factors for two modes with different cavity lengths *h* at TM polarization.

Mode type	*h* (nm)	Γ

mode 1	700	0.5381
	900	0.4806
	1200	0.3782
mode 2	700	0.6344
	900	0.7157
	1200	0.7513

As discussed above, we can obtain a high-*Q*-factor resonance by reducing α. In addition, the high *Q*-factor can also be achieved by varying the cavity length *h*, which is necessary for refractive index sensing applications. When the QBIC is applied to refractive index sensing, it enables more sensitive detection owing to its high figure of merit (FOM), the physical mechanism of which uses resonant position variations to detect changes in the refractive index of the surrounding medium. In this section, we will investigate the sensing performance of the proposed structure through the variation of two key structural parameters (α and *h*). Besides the *Q*-factor, the sensitivity (*S*) and FOM are also two important parameters for a refractive index sensor, which are defined as the ratio of the resonant wavelength drift to the change in refractive index of the surrounding environment (S = Δλ_res_/Δ*n*_c_) and the ratio of sensitivity to the FWHM (FOM = S/FWHM), respectively [55].

Taking the structural parameters previously mentioned as an example, the proposed structure can be fabricated as follows [56]. At first, the gratings of the bottom layer are fabricated using electron beam lithography (EBL) and reactive ion etching (RIE) on a SOI chip with a single crystalline silicon device layer and a buried oxide (BOX), where this SOI chip serves as the receiving substrate in an adhesive bonding process. Next, another bare SOI chip was bonded to the previously fabricated recipient substrate as a donor substrate, which is spin-coated using SU-8 on both the recipient and donor substrates. The silicon handle of the donor substrate is then removed by mechanical polishing and deep RIE, followed by removal of the BOX layer of the donor substrate by wet etching using hydrofluoric acid. Finally, the gratings are fabricated on the top layer with EBL and RIE, while the silicon handle and BOX layer on top are removed in the same way. It should be pointed out that the fabrication of the device requires removing the silicon handle and BOX layer twice (it makes the structure symmetrical), which makes the fabrication challenging to some extent. However, the simulation results show that the symmetrical structure of the device can improve the performance of the sensor.

**Figure 8:**
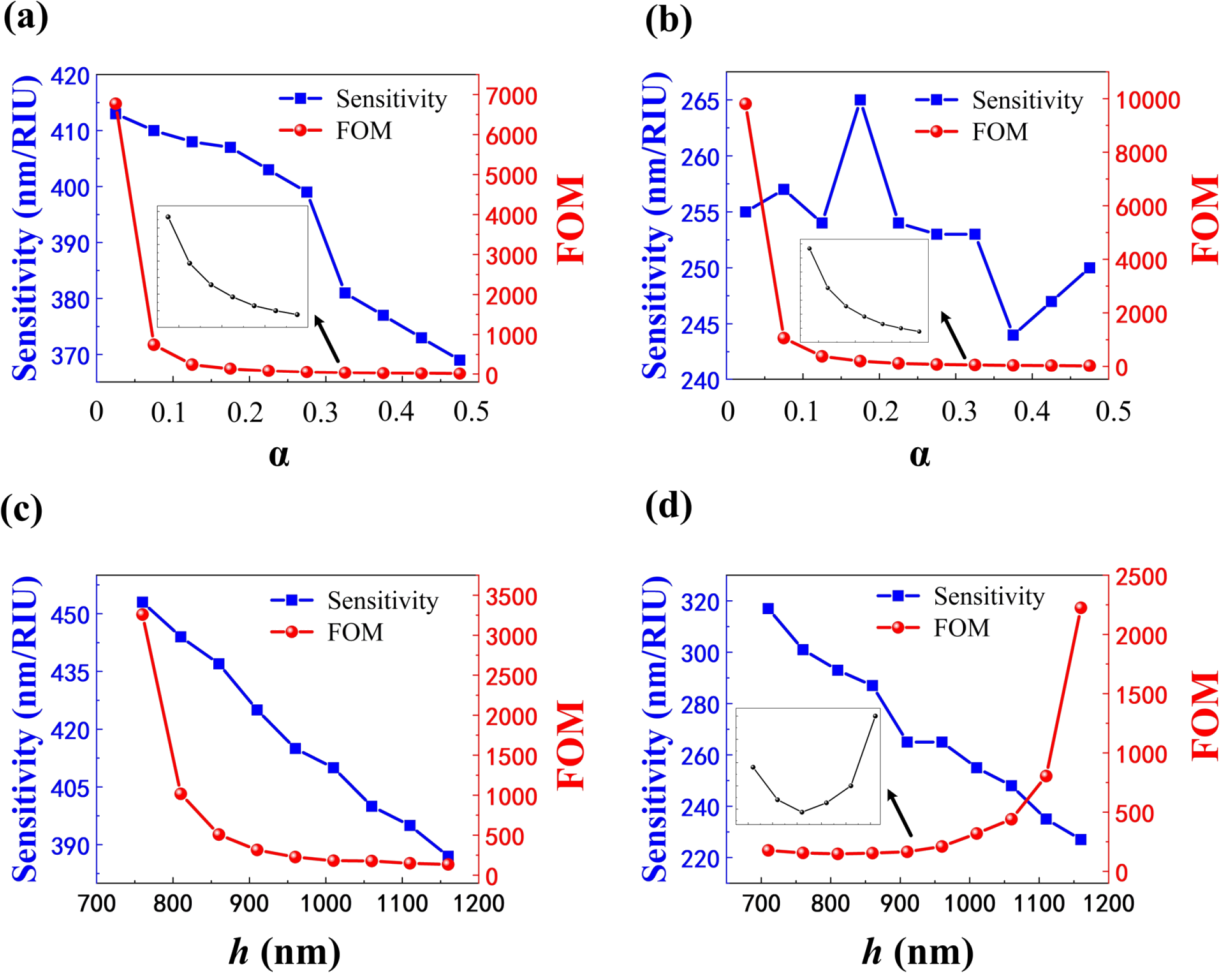
The *S* and FOM of a) mode 1 and b) mode 2 at different α values and *h* = 1000 nm. The *S* and FOM of c) mode 1 and d) mode 2 at different *h* values and α = 0.15.

The *h* value is fixed at *h* = 1000 nm and Figure 8a and Figure 8b demonstrate the *S* and FOM for the two modes with different α values. It is obvious that the FOM values of both modes decrease as α reduces. The FOM values for the two modes are 6770 and 9808 at α = 0.025, respectively. Furthermore, the highest sensitivity of the two modes is 413 and 265 nm/RIU, respectively. Here, we observe that although the FOM of mode 1 is lower than that of mode 2, which can be found from the normalized magnetic field distribution diagram that the light of mode 1 leaks more into vacuum (Figure 3b and Figure 3c), light is relatively less trapped inside the cavity, leading to more sensitivity to the refractive index change of the surrounding medium and thus higher sensitivity compared to mode 2. We then choose α = 0.15 to investigate the effect of the cavity length *h* on the sensing performance, as presented in Figure 8c and Figure 8d. It can be found that the sensitivity of both modes decreases with the increase in *h*. This is because an increase in *h* increases light trapping inside the cavity, decreasing light–matter interaction and sensitivity. On the contrary, the FOM values of the two modes do not have the same trend with increasing *h*. This is mainly attributed to the fact that the change in the cavity length of the Fabry−Pérot cavity has different magnetic field distributions for the two modes (the insets in Figure 7). The *S* is physically determined by the spatial overlap between the analyte and the evanescent waves, while the FOM is proportional to the *Q*-factor, which ultimately represents the ability of the sensor to track small changes in the refractive index of the environment [39,57].

We also found that the *S* of mode 1 at different α and *h* values follows the same trend as the FOM. The simulation results show that the values of α and *h* need to be decreased as much as possible to obtain high sensitivity and FOM of mode 1. The results were slightly different from those of mode 2, where one of the most sensitive regions exists, as shown in Figure 8b. This is may be due to the insignificant phase difference in the DLSG structure considered here [58]. Therefore, we can optimize the sensing performance by controlling the structural parameters, such as α and *h*, which are essentially the result of perturbing the artificial BIC and FP-BIC. The high sensitivity and FOM can be obtained thanks to the nearly symmetrical structure in vacuum which allows the optical field to be uniformly located in the grating and environment layers. This way, the confinement ability of the grating to the optical field is achieved while enhancing the relationship between light and matter.

Finally, the performance of the DLSG-based sensor is analyzed by choosing the structural parameters as an example: Λ = 800 nm, *h* = 1000 nm, *w* = 200 nm, α = 0.025 (the spacing between the two RDGs in a period is 205 nm). The *Q*-factors of mode 1 and mode 2 are 2.01 × 10^4^ and 4.19 × 10^4^, respectively. We summarized the *S* and FOM of QBIC when utilized for sensing as reported in other references in Table 2. One can find that although the *S* in this work is lower than that of some reported ones, it has a higher FOM and supports dual-band resonance.

**Table 2:** QBIC-based sensor performances. The description indicates the resonant mode in the reference.

Reference	Description	*S* (nm/RIU)	FOM

[3]	single-mode	657	9112
[16]	single-mode	221	4420
[25]	dual-band	526 312	8092 3387
[41]	dual-band	680 1143	183.8 317.5
this work	dual-band	413 255	6770 9808

**Figure 9:**
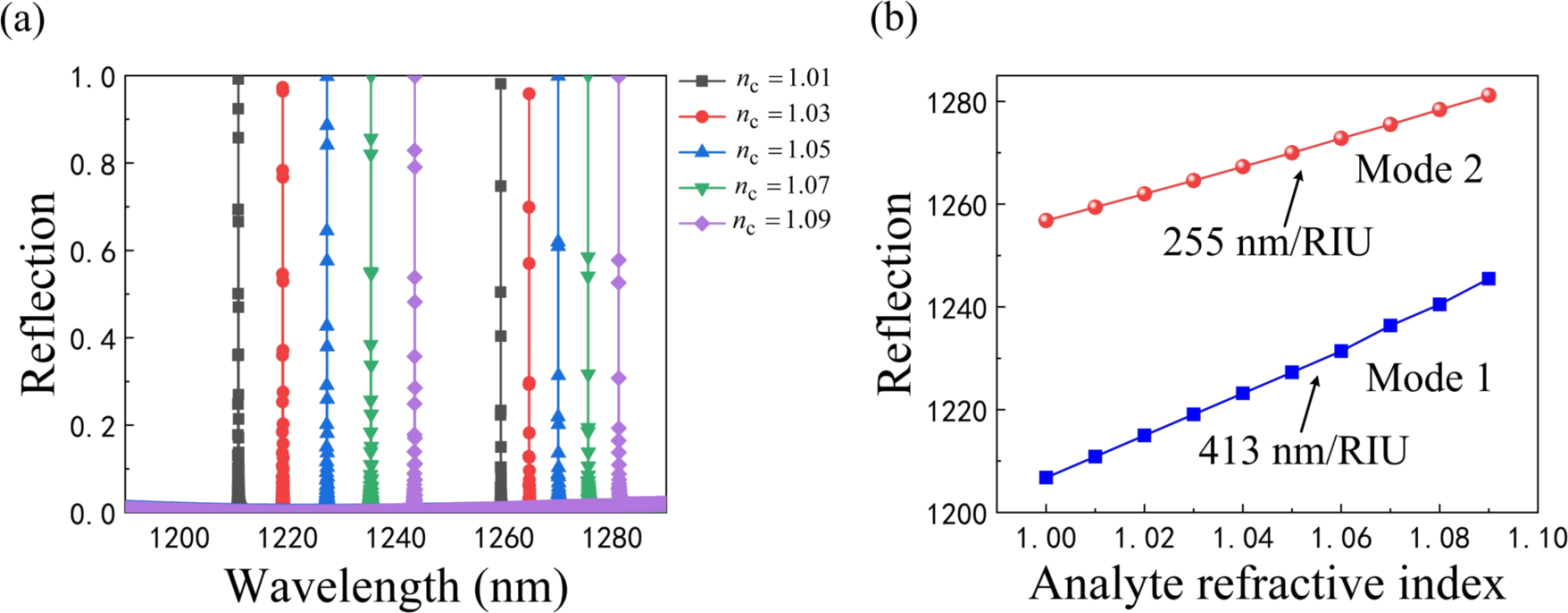
a) Reflection spectra of the sensor at different refractive indices of the gas medium. b) Positions of the two-mode reflection resonance peaks.

Figure 9a shows the simulated reflection spectra of the proposed sensor when placed in a gas medium with different refractive indexes from 1.01 to 1.09 under a TM-polarized incident plane wave. The sharp reflection peaks of both mode 1 and mode 2 show a significant red shift, despite a small fluctuation (Δ*n*_c_ = 0.02). By extracting the positions of the reflection peaks of the two modes from Figure 9a and plotting them as a function of the refractive index of the analyte, the fitted curves exhibit a good linearity as seen in Figure 9b.

## Conclusion

In summary, a DLSG-based structure is proposed, which can support dual-band high-*Q* resonances governed by BIC. Particularly, the evolution of single and double resonances can be controlled by changing the spacing of the RDGs. The artificial BIC and FP-BIC can be induced by optimizing two key structural parameters (the spacing of two gratings in a unit cell and cavity length). Specifically, we calculated the reflection spectra using RCWA for different grating spacings and cavity lengths, respectively, with the artificial BIC and FP-BIC exhibiting vanishing linewidths in the spectra. The complex eigenfrequencies of the BIC induced by different parameters were then analyzed using FEM and the corresponding radiative *Q*-factors were calculated. The two BICs were perturbed to convert an infinite *Q*-factor (ideal BIC) into a finite but high *Q*-factor (QBIC), which exhibited a dual-band high-*Q* resonance when used for optical sensing, allowing for more sensitive detection. The designed resonator has potential prospects for applications such as multi-wavelength sensing and nonlinear optics, which are important for practical nano-optics utilization.
